# Cost-Effectiveness of HIV Screening in STD Clinics, Emergency Departments, and Inpatient Units: A Model-Based Analysis

**DOI:** 10.1371/journal.pone.0019936

**Published:** 2011-05-20

**Authors:** Vimalanand S. Prabhu, Paul G. Farnham, Angela B. Hutchinson, Sada Soorapanth, James D. Heffelfinger, Matthew R. Golden, John T. Brooks, David Rimland, Stephanie L. Sansom

**Affiliations:** 1 Division of Global HIV/AIDS, Center for Global Health, Centers for Disease Control and Prevention (CDC), Atlanta, Georgia, United States of America; 2 Division of HIV/AIDS Prevention, National Center for HIV/AIDS, Viral Hepatitis, STD, and TB Prevention, Centers for Disease Control and Prevention (CDC), Atlanta, Georgia, United States of America; 3 San Francisco State University, San Francisco, California, United States of America; 4 Public Health-Seattle and King County STD Clinic and the Center for AIDS and STD, University of Washington, Seattle, Washington, United States of America; 5 Medical Specialty Service Line (111-RIM), Veterans Affairs Medical Center, Decatur, Georgia, United States of America; 6 Emory University School of Medicine, Atlanta, Georgia, United States of America; University of Cape Town, South Africa

## Abstract

**Background:**

Identifying and treating persons with human immunodeficiency virus (HIV) infection early in their disease stage is considered an effective means of reducing the impact of the disease. We compared the cost-effectiveness of HIV screening in three settings, sexually transmitted disease (STD) clinics serving men who have sex with men, hospital emergency departments (EDs), settings where patients are likely to be diagnosed early, and inpatient diagnosis based on clinical manifestations.

**Methods and Findings:**

We developed the Progression and Transmission of HIV/AIDS model, a health state transition model that tracks index patients and their infected partners from HIV infection to death. We used program characteristics for each setting to compare the incremental cost per quality-adjusted life year gained from early versus late diagnosis and treatment. We ran the model for 10,000 index patients for each setting, examining alternative scenarios, excluding and including transmission to partners, and assuming HAART was initiated at a CD4 count of either 350 or 500 cells/µL. Screening in STD clinics and EDs was cost-effective compared with diagnosing inpatients, even when including only the benefits to the index patients. Screening patients in STD clinics, who have less-advanced disease, was cost-effective compared with ED screening when treatment with HAART was initiated at a CD4 count of 500 cells/µL. When the benefits of reduced transmission to partners from early diagnosis were included, screening in settings with less-advanced disease stages was cost-saving compared with screening later in the course of infection. The study was limited by a small number of observations on CD4 count at diagnosis and by including transmission only to first generation partners of the index patients.

**Conclusions:**

HIV prevention efforts can be advanced by screening in settings where patients present with less-advanced stages of HIV infection and by initiating treatment with HAART earlier in the course of infection.

## Introduction

More than 1.1 million people in the U.S. are living with human immunodeficiency virus (HIV) infection, of whom approximately one fifth are undiagnosed and unaware of their infection.[1] Identifying persons unaware of their infections as early as possible is a national public health priority.[2] Transmission rates for persons unaware of their HIV infection are estimated to be more than three times the rates for persons aware of their infection.[3] There are also substantial benefits for the health of HIV-infected persons and reduced transmission of HIV to uninfected persons associated with the early initiation of highly active antiretroviral therapy (HAART) for those aware of their serostatus.[4]–[6]

The Centers for Disease Control and Prevention (CDC) and other public health agencies have promoted HIV testing in sexually transmitted disease (STD) clinics in the U.S. for almost two decades. To increase early diagnosis, the CDC now recommends that “diagnostic HIV testing and opt-out HIV screening be a part of routine clinical care in all health-care settings,” such as hospital emergency departments (EDs) and outpatient clinics.[7] HIV diagnostic testing of hospitalized persons with opportunistic infections or other findings suggestive of HIV has been widely available since the mid-1980s.

Previous cost-effectiveness analyses of HIV testing have shown that population-based screening protocols are cost-effective except when there is very low HIV prevalence.[8] [9], [10] These results were derived from analyses of the HIV epidemic in the U.S. that focused on screening at various intervals and that incorporated national data on HIV prevalence and incidence combined with plausible assumptions about background testing and detection of HIV through case finding. However, there is a paucity of research comparing the costs and effectiveness of diagnosing HIV infection in specific settings where persons vary in the stage of their disease at the time of testing.[11]

Recent literature indicates that early initiation of HAART may be both effective and cost-effective in preventing and treating HIV.[4]–[6], [12]–[16] In December, 2009 the Department of Health and Human Services Panel on Antiretroviral Guidelines recommended antiretroviral therapy for patients with CD4 counts up to 500 cells/µL, and half the panel recommended starting therapy at higher CD4 counts.[6] These recommendations were based on a reduction in AIDS-related mortality from earlier initiation of HAART found in observational research studies. Clinical studies have shown that viral load suppression through antiretroviral therapy may also delay or prevent some non-AIDS-defining complications including kidney, liver, and cardiovascular disease.[6] HIV modeling studies have suggested that universal voluntary HIV testing and immediate implementation of HAART (“test and treat” strategies) could have a major impact on the HIV epidemic through a reduction in viral load and decreased transmission.[17], [18]

In this study, we evaluate the cost-effectiveness of HIV testing based on the CD4 cell count at diagnosis. To do this, we use illustrative examples comparing routine screening in STD clinics in urban areas with a large population of men who have sex with men (MSM); routine screening in hospital EDs; and diagnostic testing based on clinical manifestations of HIV infection in inpatient units. Routine screening is a process where age-eligible persons are offered point-of-care rapid HIV testing in accordance with CDC's revised recommendations for HIV testing in health care settings.[7] Two of the settings, STD clinics and EDs, were emphasized in these recommendations.[7] HIV testing is common in STD clinics, and some EDs have begun pilot programs for HIV screening.[19]–[21] Most of the literature on screening in EDs has focused on the feasibility and acceptability of these procedures. Including the effects of both disease progression and transmission, we compare the cost-effectiveness of testing in these settings to each other and to diagnostic, inpatient testing. We examine both the case of initiating treatment of HIV-infected patients with HAART at a CD4 count of 350 cells/µL and 500 cells/µL. The analysis accounts for program costs, treatment-related costs, and health outcomes of both the individuals diagnosed with HIV in these settings and the partners they infect with HIV.

## Methods

### Model

We developed the Progression and Transmission of HIV/AIDS (PATH) model to estimate the quality-adjusted life expectancy and costs of persons diagnosed with HIV infection at various stages of the disease. PATH is an individual Monte Carlo simulation health state transition model that tracks index patients through different phases of HIV from infection until death. It also includes transmission and follows the infected partners of the index patients until death. The model was developed in Microsoft Excel (Version 2003, Microsoft Corporation, Redmond, WA) with Visual Basic Applications (Version 6.3, Microsoft Corporation, Redmond, WA). Distributions, random numbers, and simulations were generated with @Risk (Version 4.5.7, industrial edition, Palisade Corporation, Ithaca, NY). The unit of time progression is a three-month period representing a quarter of a calendar year, with costs, quality-adjusted life years (QALYs) lost, and other outcomes computed for each quarter. A summary of key input parameters for the model is presented in Table 1. The assumptions, technical details of the model, schematic flowcharts, and the full set of input parameters are presented in Appendix S1, The Progression and Transmission of HIV/AIDS (PATH) Model.

**Table 1:** Summary of Input Parameters.

Variable	Base Case Value	Range	Source
**Natural Disease Progression**			
CD4 cell count when infected (cells/µL)	900	750–900	[47]
HIV viral load set point (log_10_ copies/ml)	4.5	4.0–5.0	[48], [49]
Cumulative quarterly probability of developing an opportunistic infection (%)	0.3–35.3^1^		[8], [50]
**HAART Regimens**			
Minimum CD4 cell count to initiate HAART (cells/µL)	350/500		[6]
Suppressed HIV viral load level (log_10_ copies/ml)	1.3	1.0–2.7	[51]
Rebound HIV viral load level (log_10_ copies/ml)	3.7	3.1–4.5	[52]
Maximum number of HAART regimens	4		2
Probability of virologic suppression in HAART regimens 1–4	0.80		2
**Quarterly Costs (2009 $)**			[35]
Inpatient and outpatient resource utilization	905–6,007^3^		
Additional costs of opportunistic infections (each occurrence)	3,492–20,542^4^		
Additional cost of HAART (each quarter)	4,143–13,699^5^		
**Annual Rates of Sexual Transmission (# events per year per person)**			[38], [39]
Acute	0.751		
Non-acute unaware	0.093		
Non-acute aware, not on HAART	0.041		
Non-acute aware, on HAART	0.008		
**Other Variables**			
Age at infection (years)	35	30–40	6
Discount rate for costs and quality-adjusted life years (QALYs)	3%		[37]
Utility weights to estimate quality-adjusted life years (QALYs)	.935–.702^7^		[33]

1 The lower and upper bounds reflect probabilities for CD4 cell counts of >500 cells/µL and 0–50 cells/µL, respectively.

2 Expert opinion (2009).

3 Costs vary by CD4 cell count, HAART usage, and history of AIDS-defining opportunistic infection.

4 These numbers represent costs for different opportunistic illnesses.

5 The lower and upper bounds reflect costs for the first and fourth HAART regimens. Costs for the other regimens lie in between these values.

6 Written communication, R. Song, Centers for Disease Control and Prevention, June, 2008.

7 Utility weights vary by CD4 cell count and presence of opportunistic infection.

We created three scenarios — one each for routine screening in STD clinics, routine screening in hospital EDs, and diagnoses made in inpatient settings. We ran the PATH model for 10,000 iterations for each scenario. Each iteration represented an individual, or an index person, whom we tracked from infection until death. The three scenarios differed only in CD4 cell count at diagnosis, undiagnosed seropositivity rate, associated screening costs, and assumptions made about the proportion of newly diagnosed persons who were linked to care.

### Test settings and patient populations

For STD clinics, we assumed that persons visit a clinic for sexual education, health examinations, tests, and treatments, and that screening with a rapid HIV test is routinely conducted as a part of a program for STD prevention. We based our analysis on clinics located in an urban area with a large MSM population in which many persons are tested frequently. For diagnosis in an ED, we assumed that people visit an ED facility because they need urgent or emergency medical care and are routinely screened with a rapid test. For HIV diagnosis in inpatient facilities, we assumed that physicians conduct diagnostic testing (e.g., order HIV tests based on the clinical manifestations of patients) using conventional testing with an HIV enzyme immunoassay (EIA) of serum obtained by venipuncture. In all three settings, positive EIA and rapid tests were assumed to be confirmed with a Western blot.

For CD4 cell count at diagnosis in STD clinics, we used data from the One-on-One program of the Public Health – Seattle & King County (PHSKC) STD Clinic in Washington state from January 2006 to June 2008 (Table 2).(Written communication, M Golden, Public Health-Seattle & King County STD clinic and the Center for AIDS & STD, University of Washington, Seattle, May, 2009. See also [22]) The One-on-One program refers people diagnosed with HIV at Seattle and King County public health clinics to treatment. This clinic is representative of a testing program in an urban area with a large MSM population where the clients are tested with increasing frequency.[23] For the MSM tested, the median CD4 cell count at diagnosis was 429 cells/µL (range 5–1,287 cells/µL).

**Table 2:** Input Parameters That Vary With Settings.

Setting	Median CD4 Cell Count at Diagnosis (cells/µL)	Undiagnosed Seropositivity Rate in the Setting (%)	Cost of a Positive HIV Test/Negative HIV Test (2009 $)^1^	Total Program Cost per HIV-Infected Person (2009 $)	Linkage to Care Assumptions
**Inpatient (diagnostic testing)**	36	14.3[53]	62.4/5.3	94.1	100% following diagnosis
Range^2^	2–847				
Sample size^3^	69[24]				
**Emergency department (screening)**	356	0.7[19]	73.4/16.5	2,413.50	65% following diagnosis; 15% when CD4 cell count = 200 cells/µL; 20% as inpatients
Range	4–1,020				
Sample size	55[19]				
**Sexually transmitted disease clinic (screening)**	429	0.8[54] 4	85.4/19.7	2,527.50	65% following diagnosis; 15% when CD4 cell count = 200 cells/µL; 20% as inpatients
Range	5–1,287				
Sample size	398[54] 4				

1 Test costs were derived from [36].

2 Range of CD4 cell count values in the source study.

3 Number of persons diagnosed in the source study.

4 Also, written communication with M. Golden, Public Health-Seattle & King County STD Clinic and the Center for AIDS & STD, University of Washington, Seattle, May, 2009.

For CD4 cell count at diagnosis in EDs, we used the results from a program of expanded HIV screening and on-site rapid testing primarily among adult Hispanic and non-Hispanic black patients in an urban academic ED in Oakland, CA (Table 2) (median CD4 count 356 cells/µL; range 4–1,020 cells/µL).[19]

For CD4 cell count at diagnosis in inpatient facilities, we used data on inpatients discharged with a new diagnosis of HIV or AIDS at two academic medical centers in Boston, MA (Table 2) (median CD4 cell count at diagnosis 36 cells/µL; range 2–847 cells/µL).[24] These data are consistent with other studies.(Written communication, D. Rimland, VA Medical Center, Decatur, GA, August, 2009. See also [25])

### Linkage to care

We assumed that all patients diagnosed in the inpatient setting were linked to care in the quarter following diagnosis. For patients diagnosed in the ED and STD settings, we assumed that 65% were linked to care in the quarter following diagnosis, and that an additional 15% were linked to care by the time their CD4 cell count decreased to 200 cells/µL. The remaining 20% were assumed to be diagnosed as inpatients and were linked to care when their CD4 cell count decreased to 36 cells/µL, the median CD4 cell count at diagnosis in inpatient facilities.[24] These assumptions are consistent with data from studies of linkage to care in various settings.[26]–[29]

### Disease progression

We included the following phases of HIV infection as health states in the PATH model: acute infection, asymptomatic HIV infection, symptomatic HIV infection or acquired immunodeficiency syndrome (AIDS), and death. CD4 cell count and HIV viral load were the key determinants of disease progression in this model. When individuals were linked to care according to the above assumptions, they became eligible for HAART when their CD4 cell count decreased to a threshold of either 350 or 500 cells/µL to model previous and current treatment guidelines.[6], [30] Persons linked to care with higher CD4 cell counts did not initiate HAART until their CD4 cell count decreased to these thresholds, whereas persons linked to care with CD4 cell counts under the thresholds initiated HAART in the quarter following diagnosis. The PATH model included up to four suppressive HAART regimens followed by a single salvage non-suppressive regimen.

### Life expectancy and QALYs lost to infection

We predicted the probability of death during each quarter following diagnosis based on probabilities related to age and CD4 count at initiation of antiretroviral treatment.[31], [32] We assigned a utility weight ranging from 0 (for death) to 1 (for perfect health) to an individual's health state for each quarter survived, based on that individual's CD4 cell count during that quarter and whether the individual had an opportunistic illness (OI) such as *Pneumocystis jiroveci* pneumonia (PCP). We used quality-of-life weights from Tengs and Lin[33] and aggregated them over the person's life. We then subtracted this sum from the QALYs associated with an HIV-uninfected person, assuming a QALY of 1 (good health) for the entire life expectancy from the age of HIV infection,[34] to estimate the QALYs lost due to HIV infection. Measuring QALYs lost due to infection resulted in consistent quality of life estimates when transmissions to partners were included in the model. A decrease in QALYs lost in one setting compared with QALYs lost in another represents a gain in QALYs in the first setting.

### Costs

We included both treatment costs and program costs in 2009 dollars estimated from the provider perspective. Treatment costs, derived from lifetime cost estimates by Schackman et al.,[35] included the costs of health care resource utilization, antiretroviral therapy, laboratory monitoring (i.e., CD4 cell count, HIV viral load determination, and genotypic antiretroviral resistance testing), diagnosing and treating OIs, and costs incurred during the last month of life.

To estimate program costs, we calculated only the marginal costs associated with testing and counseling in a particular setting for both HIV-infected and uninfected persons. We assumed that the settings evaluated already had HIV testing ability, so fixed and start-up costs were not included in our calculations. For inpatient facilities, we estimated the laboratory costs for conventional HIV testing and post-test counseling costs only for HIV-infected persons. For the ED and STD clinic settings, we included additional costs associated with rapid HIV testing, such as the costs for collecting specimens, the test kits, and post-test counseling for infected persons. We did not include the cost of administrative overhead and other costs that would have been incurred in the absence of a screening program. These program costs were varied in the sensitivity analysis, particularly for STD clinics, to reflect the repeat testing by MSM that often occurs in these settings.[23]

To include the costs of persons who are tested but are not HIV infected, we computed program costs per HIV-infected person identified using the following formula for each setting: {[p * Cost _HIV+_ + (1−p) * Cost _HIV−_]/*p*} where *Cost_ HIV+_*  =  cost of testing an HIV-infected person, *Cost _HIV−_*  =  cost of testing an uninfected person, and *p*  =  the undiagnosed HIV seropositivity rate in that particular setting. For example, in inpatient settings the total cost per HIV-infected person derived from Table 2 data equals [(0.143) * 62.4+(1−0.143) * 5.3)]/0.143 = $94.1.

*Cost _HIV+_* and *Cost _HIV−_* for a particular setting were derived from estimates by Farnham et al.[36] (Table 2). We discounted future costs and QALYs at a rate of 3% per year[37] from the quarter of infection.

### Disease transmission

We used a quarterly probability of HIV transmission per infected individual derived from an annual transmission rate to add HIV transmission from index patients to the model (Table 1). We estimated transmission probabilities on the basis of a model, first developed by Pinkerton[38] and later updated by Prabhu et al.[39], which is explained in Appendix S1. Transmission probabilities were derived for those acutely infected and unaware of their infection, those non-acutely infected and unaware, and for those non-acutely infected who were aware and either not on or on a HAART regimen. We used separate rates for sexual and injection drug use (IDU) transmission, and we assumed that 12.9% of the index persons were IDU in all settings.[40]

We evaluated secondary transmissions for a single generation of transmissions, i.e., transmission of HIV from index persons to their partners. We assumed that all partners who acquired infections from an index patient were diagnosed at a CD4 cell count of 500 cells/µL and were linked to care based on assumptions similar to those for persons diagnosed in ED and STD clinics. We standardized the linkage to care and treatment approach for infected partners because our primary interest is assessing the timing of diagnosis, linkage to care, and initiation of treatment of index patients on the cost-effectiveness of HIV diagnosis in different settings.

### Measuring cost-effectiveness

We estimated the costs (treatment and program) and QALYs lost to infection for each of the 10,000 index patients for each setting, and we computed the mean costs (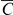) and mean QALYs lost to infection (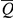) for each setting. We then used the ratio of the differences in mean costs and differences in mean QALYs to compute the incremental cost-effectiveness ratio (ICER). To calculate ICERs with transmission effects, we included the costs and QALYs lost to infection for the index persons and their infected partners. We calculated 95 percent confidence intervals for mean and incremental costs and QALYs.

Incremental cost-effectiveness ratios may be negative, indicating cost-savings resulting from an increase in QALYs gained and a decrease in costs, or positive, showing that additional costs are required to achieve the gain in QALYs. For the latter, $100,000 per QALY gained represents a reasonable current estimate of the amount society is willing to pay to gain a QALY, although this amount may be even higher.[41]–[43]

### Sensitivity analyses

The base case simulation of the model for 10,000 iterations used point estimates for all variables in the model. A simulation of 10,000 iterations was necessary as the outcomes of the model reflected the probabilities of the occurrence of different events, such as the development of an opportunistic illness or the probability of dying during the quarter after HAART had been initiated, for each of the 10,000 index persons. Values of the model variables were drawn directly from the literature as noted previously and in Appendix S1. We present base case results both excluding and including transmission and with the assumption of patients initiating HAART at a CD4 count of either 350 or 500 cells/µL.

We then performed one-way sensitivity analyses of the impact of changes in selected variables on the STD-ED ICERs in the base case, assuming initiation of HAART at a CD4 count of 350 cells/µL. These variables included the undiagnosed HIV seropositivity rate in the different testing settings, overall program costs, STD clinic program costs, HIV treatment costs, age at infection, the probability of viral load suppression, and the transmission probabilities. The differences between testing in the STD and ED settings were analyzed in more detail by varying the CD4 count at diagnosis in the STD setting. The impact of linkage to care was examined by assuming that all index patients and their partners were immediately linked to care.

To reflect the overall uncertainty in decision analytic models, we also ran a probabilistic sensitivity analysis by assigning distributions around the point estimates of key variables based on accepted conventions.[44] These variables included: age at infection; CD4 count when infected; CD4 count at diagnosis; set point viral load; the levels of suppressed, rebound, and salvage therapy viral load; and the decline in CD4 count at a specific viral load stratum. Normal distributions were used for most variables. Given the importance of CD4 count at diagnosis for this analysis and the small sample sizes in the studies reporting these values, we used the cumulative distribution based on the minimum, maximum, and inter-quartile values for these variables in an attempt to most accurately use the available data.

## Results

### Cost-effectiveness of HIV testing in different settings

#### Initiate HAART at a CD4 count of 350 cells/µL

In the base case analysis, assuming initial treatment with HAART at a CD4 count of 350 cells/µL and excluding the effects of HIV transmission (Table 3), individuals diagnosed with HIV in the ED setting gained an additional 2.5 (2.3–2.6) QALYs compared with individuals diagnosed as inpatients. Mean discounted total costs (program costs and treatment costs) incurred were $398,833 ($395,898–$401,768) for those diagnosed in ED settings and $313,655 ($310,854–$316,456) for persons diagnosed with HIV in inpatient settings. Compared to diagnosis in inpatient settings, the cost per QALY gained for a diagnosis in the ED setting was $34,597. The mean discounted total cost of diagnosing individuals in STD clinics was $399,844 ($396,909–$402,779) or $1,012 more than in the ED setting. However, the discounted QALYs lost to HIV infection were the same in both the ED and STD clinic settings because we assumed that index patients linked to care in these settings initiated HAART at the same time following infection, i.e., when their CD4 counts decreased to 350 cells/µL. Therefore, the ICER between these settings was undefined, given that the incremental costs were divided by a zero change in QALYs.

**Table 3:** Cost-Effectiveness Analysis of Testing in Different Settings, Initiate HAART at CD4 cell count = 350 cells/µL.

Setting	Mean Discounted Costs (2009 $)	Mean Discounted Quality-Adjusted Life Years Lost to Infection (QALY)	Incremental Cost	Incremental QALY Gained	Incremental Cost-Effectiveness Ratio (ICER) ($/QALY)
***Excluding Transmission***					
**Inpatient (diagnostic testing)**	313,655	7.313	–	–	–
(95% CI)^1^	(310,854–316,456)	(7.229–7.397)	–	–	–
**Emergency department (screening)**	398,833	4.851	85,178	2.462	34,597
(95% CI)	(395,898–401,768)	(4.767–4.935)	(81,121–89,235)	(2.343–2.581)	–
**Sexually transmitted disease clinic (screening)**	399,844	4.851	1,012	0.000	Undefined^2^
(95% CI)	(396,909–402,779)	(4.767–4.935)	(−3,140–5,162)	–	–
***Including Transmission***					
**Inpatient (diagnostic testing)**	817,419	14.097	–	–	–
(95% CI)	(809,196–825,642)	(13.904–14.290)	–	–	–
**Emergency department (screening)**	816,824	10.130	−595	3.967	Cost-saving^3^
(95% CI)	(808,954–824,694)	(9.958–10.302)	(−11,977–10,787)	(3.708–4.226)	–
**Sexually transmitted disease clinic (screening)**	800,716	9.866	−16,108	0.264	Cost-saving^3^
(95% CI)	(792,950–808,482)	(9.699–10.033)	(−27,164–−5,052)	(0.024–0.504)	–

1 CI = confidence interval.

2 These ratios are undefined because there is no increase in QALYs between the emergency department and sexually transmitted disease clinic settings. The incremental cost would be divided by zero.

3 Screening in the setting is cost-saving compared with screening in the previous setting because there is an increase in QALYs and a decrease in costs.

In other model results (data not shown), index patients in both the ED and STD clinic settings started HAART at a median CD4 count of 345 cells/µL, had a mean time from infection to the start of HAART of 11.2 years, were on HAART for a mean time of 25.3 years, and experienced the onset of AIDS an average of 22.0 years from the time of infection. Mean life expectancy with infection was 36.5 years, which is consistent with the literature.[45]

We estimated that persons diagnosed in STD clinics transmitted HIV to an average of 1.37 individuals compared with 1.44 individuals for those diagnosed in EDs and 1.83 individuals for those diagnosed in inpatient settings. When including the costs and QALYs gained that were associated with transmission, diagnosing persons in ED settings was found to be cost-saving compared with diagnosis in inpatient facilities (except at the upper bound of the 95% confidence interval for incremental costs). Diagnosis in STD clinics was also cost-saving when compared with ED settings and inpatient facilities (Table 3).

#### Initiate HAART at a CD4 count of 500 cells/µL

In the case excluding transmission effects where treatment with HAART for the index patient was initiated at a CD4 count of 500 cells/µL (Table 4), the cost per QALY gained for screening in the ED compared with inpatient testing was essentially the same as in Table 3. However, when comparing screening in STD clinic settings with ED screening, there was an increase of 0.4 (0.2–0.5) QALYs and an ICER of approximately $60,000 per QALY gained. The median CD4 count at initiation of HAART was 415 cells/µL for index patients screened in STD clinics compared with 345 cells/µL for those screened in EDs (results not shown). Index patients in STD clinic settings began HAART an average of 10.4 years following infection compared with 11.2 years among those screened in the ED, and they were on HAART for an average of 26.8 years compared with 25.1 years for ED index patients (data not shown). When the effects of reduced transmission were included in the analysis, screening in the ED setting remained cost-saving compared with inpatient testing (except at the upper bound of the 95% confidence interval) and screening in STD clinic settings remained cost-saving compared with ED screening (Table 4).

**Table 4:** Cost-Effectiveness Analysis of Testing in Different Settings, Initiate HAART at CD4 cell count = 500 cells/µL.

Setting	Mean Discounted Costs (2009 $)	Mean Discounted Quality-Adjusted Life Years Lost to Infection (QALY)	Incremental Cost	Incremental QALY Gained	Incremental Cost-Effectiveness Ratio (ICER) ($/QALY)
***Excluding Transmission***					
**Inpatient (diagnostic testing)**	313,520	7.331	–	–	–
(95% CI)^1^	(310,726–316,314)	(7.247–7.415)	–	–	–
**Emergency department (screening)**	396,164	4.942	82,644	2.389	34,594
(95% CI)	(393,273–399,055)	(4.859–5.025)	(78,624–86,664)	(2.271–2.507)	–
**Sexually transmitted disease clinic (screening)**	417,883	4.580	21,719	0.362	59,997
(95% CI)	(414,935–420,831)	(4.498–4.662)	(17,590–25,848)	(0.245–0.479)	–
***Including Transmission***					
**Inpatient (diagnostic testing)**	867,404	13.519	–	–	–
(95% CI)	(858,483–876,325)	(13.334–13.704)	–	–	–
**Emergency department (screening)**	859,993	9.712	−7,411	3.807	Cost-saving^2^
(95% CI)	(851,501–868,485)	(9.549–9.875)	(−19,728–4,906	(3.560–4.054)	–
**Sexually transmitted disease clinic (screening)**	856,432	8.986	−3,561	0.726	Cost-saving^2^
(95% CI)	(848,077–864,787)	(8.828–9.144)	(−15,474–8,352)	(0.499–0.953)	–

1 CI = confidence interval.

2 Screening in the setting is cost-saving compared with screening in the previous setting because there is an increase in QALYs and a decrease in costs.

### Sensitivity analyses

The results of the one-way sensitivity analyses in Table 5 comparing screening in STD clinic settings with ED screening showed that the base case results in Table 3 were robust with respect to changes in key variables in the analysis. Variations in undiagnosed HIV seropositivity rates, program costs, HIV treatment costs, age at infection, the probability of viral load suppression, and transmission rates had little impact on the STD-ED incremental cost-effectiveness ratios. These ICERs remained undefined when the transmission effects were excluded, given the zero change in QALYs between the two settings. Screening in the STD clinic setting remained cost-saving compared with ED screening when the benefits of reduced transmission were included. When the CD4 count at diagnosis in the STD setting was varied by increments of 20 cells/µL from 356 cells/µL (equal to the base case value for the ED setting) to 436 cells/µL, STD screening remained cost-saving compared with ED screening even for a difference as small as 20 cells/µL when transmission benefits were included in the analysis. Assuming 100 percent linkage to care for both index patients and partners also did not change the results of the analysis.

**Table 5:** Sensitivity Analysis, Base Case Model, Screening in Sexually Transmitted Disease (STD) Clinic Settings Versus Emergency Department (ED) Screening.

Variable	Values	Incremental Cost-Effectiveness Ratio (ICER)	
		Excluding Transmission^1^	Including Transmission^2^
Undiagnosed HIV Seropositivity			
Base Case	STD: 0.8%; ED: 0.7%	Undefined	Cost-saving
Low	STD: 0.56%; ED: 0.5%	Undefined	Cost-saving
High	STD: 3.0%; ED: 1.5%	Undefined	Cost-saving
Program Cost	Adjustment Factor		
Base Case	1.0	Undefined	Cost-saving
Low	0.5	Undefined	Cost-saving
High	2.0	Undefined	Cost-saving
Program Cost: STD Clinic Only	Adjustment Factor		
Base Case	1.0	Undefined	Cost-saving
Low	0.5	Undefined	Cost-saving
High	2.0	Undefined	Cost-saving
Treatment Cost	Adjustment Factor		
Base Case	1.0	Undefined	Cost-saving
Low	0.8	Undefined	Cost-saving
High	1.2	Undefined	Cost-saving
Age			
Base Case	35	Undefined	Cost-saving
Low	30	Undefined	Cost-saving
High	40	Undefined	Cost-saving
Probability of Viral Load Suppression			
Base Case	0.80	Undefined	Cost-saving
Low	0.72	Undefined	Cost-saving
High	0.88	Undefined	Cost-saving
Annual Rates of Transmission			
Base Case		Undefined	Cost-saving
Reduce by 25%		Undefined	Cost-saving
Reduce by 50%		Undefined	Cost-saving
STD Clinic CD4 Cell Count at Diagnosis (cells/µL)			
356 (same as ED)		Undefined	Undefined
376		Undefined	Cost-saving
396		Undefined	Cost-saving
416		Undefined	Cost-saving
436		Undefined	Cost-saving
Linkage to Care			
Base Case (65%, 15%, 20%)		Undefined	Cost-saving
100%		Undefined	Cost-saving

1 These ratios are undefined because there is no increase in QALYs between the ED and STD clinic settings. The incremental cost would be divided by zero.

2 Screening in the STD clinic setting is cost-saving compared with screening in the ED setting because there is an increase in QALYs and a decrease in costs.

In sensitivity analysis (data not shown), the ED-inpatient ICERs were all in the same range as for the base case. Thus, the results for all the incremental cost-effectiveness ratios were robust in the sensitivity analysis.

When the model was run with a probabilistic sensitivity analysis around key variables (Table 6), excluding transmission effects and assuming treatment with HAART at a CD4 count of 350 cells/µL, the ED-inpatient incremental cost-effectiveness ratio was approximately the same as in the base case (Table 3). However, the STD-ED ICER was $44,000/QALY gained compared with the undefined STD-ED ICER in the base case (Table 3). When transmission benefits were included in the analysis, initiating treatment with HAART at a CD4 count of either 350 or 500 cells/µL (Tables 6 and 7) was cost-saving for both the ED-inpatient and STD-ED comparisons (except at the upper bound of the 95% confidence interval).

**Table 6:** Cost-Effectiveness Analysis of Testing in Different Settings, Probabilistic Sensitivity Analysis, Initiate HAART at CD4 cell count = 350 cells/µL.

Setting	Mean Discounted Costs (2009 $)	Mean Discounted Quality-Adjusted Life Years Lost to Infection (QALY)	Incremental Cost	Incremental QALY Gained	Incremental Cost-Effectiveness Ratio (ICER) ($/QALY)
***Excluding Transmission***					
**Inpatient (diagnostic testing)**	334,003	7.573	–	–	–
(95% CI)^1^	(330,517–337,489)	(7.468–7.678)	–	–	–
**Emergency department (screening)**	401,807	5.506	67,804	2.067	32,803
(95% CI)	(398,584–405,030)	(5.413–5.599)	(63,056–72,552)	(1.927–2.207)	–
**Sexually transmitted disease clinic (screening)**	409,952	5.320	8,145	0.186	43,790
(95% CI)	(406,744–413,160)	(5.228–5.412)	(3,598–12,692)	(0.056–0.316)	–
***Including Transmission***					
**Inpatient (diagnostic testing)**	794,190	13.491	–	–	–
(95% CI)	(785,663–802,717)	(13.296–13.686)	–	–	–
**Emergency department (screening)**	793,861	10.330	−329	3.161	Cost-saving^2^
(95% CI)	(785,864–801,858)	(10.157–10.503)	(−12,019–11,361)	(2.900–3.422)	–
**Sexually transmitted disease clinic (screening)**	783,900	9.896	−9,961	0.434	Cost-saving^2^
(95% CI)	(776,056–791,744)	(9.727–10.065)	(−21,163–1,241)	(0.192–0.676)	–

1 CI = confidence interval.

2 Screening in the setting is cost-saving compared with screening in the previous setting because there is an increase in QALYs and a decrease in costs.

**Table 7:** Cost-Effectiveness Analysis of Testing in Different Settings, Probabilistic Sensitivity Analysis, Initiate HAART at CD4 cell count = 500 cells/µL.

Setting	Mean Discounted Costs (2009 $)	Mean Discounted Quality-Adjusted Life Years Lost to Infection (QALY)	Incremental Cost	Incremental QALY Gained	Incremental Cost-Effectiveness Ratio (ICER) ($/QALY)
***Excluding Transmission***					
**Inpatient (diagnostic testing)**	339,830	7.498	–	–	–
(95% CI)^1^	(336,301–343,359)	(7.393–7.603)	–	–	–
**Emergency department (screening)**	415,374	5.356	75,544	2.142	35,268
(95% CI)	(412,053–418,695)	(5.263–5.449)	(70,698–80,390)	(2.002–2.282)	–
**Sexually transmitted disease clinic (screening)**	427,799	5.119	12,425	0.237	52,427
(95% CI)	(424,494–431,104)	(5.028–5.210)	(7,740–17,110)	(0.107–0.367)	–
***Including Transmission***					
**Inpatient (diagnostic testing)**	854,757	12.990	–	–	–
(95% CI)	(845,609–863,905)	(12.800–13.180)	–	–	–
**Emergency department (screening)**	853,593	9.808	−1,164	3.182	Cost-saving^2^
(95% CI)	(844,936–862,250)	(9.641–9.975)	(−13,759–11,431)	(2.929–3.435)	–
**Sexually transmitted disease clinic (screening)**	839,551	9.285	−14,042	0.523	Cost-saving^2^
(95% CI)	(830,981–848,121)	(9.125–9.445)	(−26,223–−1,861)	(0.292–0.754)	–

1 CI = confidence interval.

2 Screening in the setting is cost-saving compared with screening in the previous setting because there is an increase in QALYs and a decrease in costs.

## Discussion

Although individuals should always be tested when they present with clinical manifestations in inpatient settings, HIV prevention efforts can be improved by screening in settings where people present with less-advanced stages of HIV infection and by initiating treatment with HAART at those earlier disease stages. Our results illustrate the cost-effectiveness of testing for HIV infection in settings where diagnosis at higher CD4 counts early in the course of disease is likely to occur and when treatment with HAART is initiated earlier in the course of infection.

If HAART is initiated at a CD4 count of 350 cells/µL, early diagnosis is cost-effective for index patients when comparing either the ED or STD clinic setting with inpatient diagnosis. Although the mean discounted program and treatment costs were higher in the ED and STD clinic settings compared with inpatient diagnosis because patients were on HAART regimens for longer periods, there were reduced QALYs lost to HIV infection due to the delayed onset of AIDS that resulted in incremental cost-effectiveness ratios of less than $100,000 per QALY gained.[41]–[43] When the effects of transmission were included in the analysis, screening in the ED and STD clinic settings was cost-saving compared with inpatient testing.

In the base case analysis excluding transmission effects, diagnosis of index patients in STD clinics compared with the ED setting involved slightly higher costs because the earlier average diagnosis in STD clinics at a median CD4 count of 429 cells/µL (compared with 356 cells/µL in the ED setting) resulted in monitoring costs for an additional duration for the index patients. However, index patients in both settings were assumed to initiate a HAART regimen only when their CD4 counts decreased to 350 cells/µL. This fact accounted for the lack of differences in the disease progression variables, e.g., mean time from infection to start of HAART and mean time on HAART, for index patients in the STD clinic and ED settings and for the identical QALYs lost to infection in both settings.

However, earlier diagnosis in the STD clinic setting compared with the ED setting implies that index patients spend less time unaware of their serostatus in the non-acute phase of HIV infection, resulting in fewer transmissions per person. The costs of treating HIV infection comprise approximately 99% of the total costs associated with each setting. Even a small change in the number of transmissions per index patient (1.37 in STD clinics compared with 1.44 in EDs and 1.83 in the inpatient setting) results in significant treatment costs averted and makes screening in the ED setting cost-saving compared with inpatient diagnosis and screening in STD clinics cost-saving compared with the ED setting.

Thus, the cost-effectiveness issues change fundamentally when the benefits of reduced transmission are included in the model. Earlier diagnosis averts more secondary infections from the index patients. This outcome results from the modeled reduction in risky behavior following diagnosis and reduced transmission due to HIV viral load suppression achieved with HAART. These transmission effects resulted in a reduced number of secondary infections and reduced total costs (i.e., the combined costs of HIV infection for the index patient and their infected partners). Thus, settings where individuals were diagnosed earlier in their infections were cost-saving compared to settings with later diagnosis when transmission effects were included. These transmission benefits occurred even when there were very small differences in CD4 counts between index patients in the ED and STD clinic settings, given the treatment costs saved.

The analysis also changed when it was assumed that initiation of HAART began at a CD4 count of 500 cells/µL. Screening index patients in STD clinic settings was now cost-effective compared with ED settings because treatment for more patients began immediately when they were diagnosed with HIV, reducing the quality-adjusted life expectancy lost to HIV infection. Early treatment with HAART suppresses viral load, increases the patient's CD4 count and the maximum CD4 count attainable, and lowers the rate of CD4 count decline. All of these factors lower the probability of death for patients on HAART compared with HAART-naïve patients.

In our base case analysis, in which we assumed that all individuals in each setting were tested at the median CD4 count for that setting, 429 cells/µL for STD clinics, 356 cells/µL for EDs, and 36 cells/µL for inpatient settings, there were no changes in QALYs between the ED and STD clinic settings (Table 3), given that index patients in both the ED and STD clinics initiated HAART at the same time following infection, i.e., when their CD4 counts decreased to 350 cells/µL. When we drew values from cumulative distributions around the median CD4 counts at diagnosis in the different settings in the probabilistic sensitivity analysis, screening of index patients in STD clinics became cost-effective compared with ED diagnosis (an ICER of $44,000 per QALY in Table 6). Due to the nature of these distributions, individuals were tested in both settings at CD4 counts higher and lower than the median. For example, model results (not presented) showed that 25% of individuals in the ED setting were diagnosed at CD4 counts of 185 cells/µL or less compared with 309 cells/µL for STD clinics. Thus, individuals in the ED would, on average, have had a much more advanced disease stage at diagnosis compared with those diagnosed in the STD clinic setting, although both would be referred to treatment immediately after diagnosis. Therefore, if individuals in the STD clinic and ED settings are actually tested at CD4 counts that vary widely from the median, there can be a benefit to the index patients of testing and initiating HAART, on average, earlier in STD clinics than in emergency departments.

### Limitations of the Analysis

Our work is subject to a number of limitations. Data regarding disease status (CD4 cell count and HIV viral load at diagnosis) for the different HIV testing settings are very limited. In particular, the data we used for CD4 cell count at diagnosis were drawn from observations at a small number of locations. We, therefore, may not be able to generalize our findings to all EDs, STD clinics, and inpatient settings. Our analysis indicates that more data, particularly on CD4 count at diagnosis by setting, would be useful, given the differences between our base case results and those in the probabilistic sensitivity analysis where we allowed the CD4 count at diagnosis to vary around the median in each setting. On the other hand, our main finding, that diagnosing persons living with HIV at higher CD4 counts is cost-effective, is robust even with the limited data.

We may have under-estimated the costs for screening in STD clinics because we did not include any fixed costs and because many STD settings include clinics that strongly encourage repeat testing among their MSM clients. However, it would be inconsistent to use average costs (that include fixed costs) for STD clinics and marginal or incremental costs (that exclude fixed costs) for the ED and inpatient settings. Although repeat testing would increase STD clinic costs, we showed in the one-way sensitivity analysis that increasing STD screening costs by 100 percent did not change the results of the analysis. In a separate simulation (results not shown), we increased STD screening costs ten-fold from their base case value, and this variation also did not change the overall cost-effectiveness results of the analysis.

Data on linkage to care are sparse and may vary by subgroups in the population. The assumptions in this model are consistent with the existing literature, and our sensitivity analysis did not show any impact of changes in these assumptions. However, better data, particularly on linkage to care in different settings, will improve future modeling efforts.

The PATH model does not incorporate any measure of ongoing transmission beyond the first generation partners. Thus, we may underestimate the cost-effectiveness of early diagnosis as some additional secondary transmission might also be averted. On the other hand, some infections we consider to be averted might only be delayed. Use of a dynamic transmission model in an economic analysis could improve the estimates of the cost-effectiveness of different HIV screening programs, but would introduce additional complexity and uncertainty related to sexual mixing patterns, which are not well defined. Our estimates of the number of transmissions per index partner are consistent with those in the literature.[10], [46] Decreasing the transmission probabilities in the sensitivity analysis reduced the number of transmissions per index partner but did not affect the overall cost-effectiveness results.

### Conclusions

Our analysis with the PATH model showed that identifying persons with HIV while their CD4 counts are high is cost-effective and potentially cost-saving, when the effects of early diagnosis on transmission are considered. Although inpatient testing based on clinical manifestations of disease should always be undertaken, our results should prompt additional HIV case-finding efforts, particularly in venues such as STD clinics and emergency departments, where persons are likely to have higher CD4 counts at the time of diagnosis. The results can help guide decisions about implementing HIV screening and should be used to encourage the collection of additional data on CD4 count at diagnosis to identify more settings where persons are likely to be tested early in the course of disease. Our model also showed that initiating treatment with HAART earlier in the course of infection is cost-effective, making early diagnosis even more beneficial.

## Supporting Information

Appendix S1 The Progression and Transmission of HIV/AIDS (PATH) Model.
(DOC)
