# Supplementary material for: Cost-Effectiveness of HIV Screening in STD Clinics, Emergency Departments, and Inpatient Units: A Model-Based Analysis
Source: PLoS One. 2011 May 20;6(5):e19936. doi: 10.1371/journal.pone.0019936 (PMC3098845; doi:10.1371/journal.pone.0019936)
Supplement: Appendix S1 — The Progression and Transmission of HIV/AIDS (PATH) Model. (DOC) [file pone.0019936.s001.doc]

**Appendix S1: The Progression and Transmission of HIV/AIDS (PATH) Model**

Supplement to: **Cost-effectiveness of HIV Screening in STD Clinics, Emergency Departments, and Inpatient Units: A Model-Based Analysis**

Vimalanand S. Prabhu, PhD^1^, Paul G. Farnham, PhD^2,a^, Angela B. Hutchinson, PhD^2^, Sada Soorapanth, PhD^3^, James D. Heffelfinger, MD^2^, Matthew R. Golden MD^4^, John T. Brooks, MD^2^, David Rimland, MD^5^, Stephanie L. Sansom, PhD^2^

**Affiliations:**

^1^Division of Global HIV/AIDS, Center for Global Health, Centers for Disease Control and Prevention (CDC), Atlanta, GA 30333

^2^ Division of HIV/AIDS Prevention, National Center for HIV/AIDS, Viral Hepatitis, STD, and TB Prevention, Centers for Disease Control and Prevention (CDC), Atlanta, GA. 30333

^3^ San Francisco State University, San Francisco, CA. 94132

^4^ Public Health-Seattle & King County STD Clinic and the Center for AIDS & STD, University of Washington, Seattle, WA. 98104

^5^ Medical Specialty Service Line (111-RIM), VA Medical Center, Decatur GA. 30033, and Emory University School of Medicine, Atlanta, GA. 30333

^a^ Corresponding author

**Introduction**

The Progression and Transmission of HIV/AIDS (PATH) model estimates quality-adjusted life expectancy, transmission, and lifetime treatment costs for individuals infected with human immunodeficiency virus (HIV). PATH is an individual Monte Carlo simulation health state transition model that tracks individuals (index patients) and their infected partners from the time of infection to death. The model was developed in Microsoft Excel (Version 2003) with Visual Basic Applications (Version 6.3, Microsoft Corporation, WA.). Distributions, random numbers, and simulations were generated in @RISK (Industrial version 4.5.7, Palisade Corporation, NY.). The time unit of progression in the model is a calendar-year quarter.

The objective of this model is to compare the costs and health outcomes of HIV-infected persons diagnosed in different settings. We also examined secondary transmissions from index patients to their partners and estimated incremental cost-effectiveness ratios between settings with and without including these transmissions. The appendix focuses on the technical aspects of the model.

We describe how the model portrays the natural history of HIV disease, the administration of highly active antiretroviral therapy (HAART), knowledge of HIV status, quality of life, disease treatment costs, and HIV transmission. We also discuss the limitations of the model.

**Natural History of HIV Disease**

In the PATH model, the natural history of HIV disease was defined as disease progression in the absence of HAART. This is the course that individuals follow when they are undiagnosed and unaware of their disease. Individuals who are diagnosed, but who have not yet started HAART, also follow the natural history of HIV disease. Individuals enter the PATH model at the time of HIV infection and exit at death. It is possible that an individual may die before being diagnosed with HIV in the model.

We modeled the natural history of HIV disease in four phases: acute HIV infection, asymptomatic HIV infection, symptomatic HIV infection or acquired immunodeficiency syndrome (AIDS), and death. A schematic flowchart of disease progression is shown in Figure 1.

We assumed that individuals entered the model, i.e. were infected, at age 35 years (range 30 - 40 years) based on 2006 HIV incidence data. (Written communication, R. Song, Centers for Disease Control and Prevention, June, 2008) We assumed that HIV was transmitted through injection drug use (IDU) for 12.9% of the individuals and through sexual transmission for all others based on 2006 HIV incidence rates.[1] We did not model behavioral differences in risk groups (men who have sex with men (MSM), high-risk heterosexual, or IDU), and we did not stratify by gender or race.

**Acute HIV Infection**

Individuals enter the acute phase of HIV infection at the start of the model. This stage represents the first few weeks of infection in which HIV viral replication and shedding peak.[2] The duration of the acute phase may vary in different individuals and is characterized by peak HIV viral load about 2 weeks after infection.[3] HIV viral loads decline thereafter at a slow rate, but remain high until 6 to 8 weeks after infection,[4],[5] after which they remain relatively stable at the HIV viral load set-point.[6],[7] Because our unit of disease progression is a calendar-year quarter, we modeled the acute phase to last for one quarter and the HIV viral load to be constant during that quarter.

We modeled HIV viral load during the acute phase to be 5.3 log_10_ copies/ml (range 4.4 to 6.2 log_10_ copies/ml) on the basis of the median plasma HIV-1 RNA level within the first 30 days of infection reported by Schacker et al.[8,9] The CD4 cell count at infection was assumed to be 900 cells/µL (range 750 - 900 cells/µL).[10] We assumed that individuals with acute infection were not aware of their serostatus and would not have an AIDS-defining opportunistic illness (OI) during this phase. A complete list of all input parameters in the model is provided in Table 1.

**Asymptomatic HIV Infection**

Individuals in the model automatically transition to the asymptomatic HIV infection phase in the quarter following the acute infection phase. In the asymptomatic phase of the model, individuals do not have any AIDS-related signs or symptoms and remain unaware of their infection unless they are tested for HIV. This phase is characterized by a steady HIV viral load or “HIV viral load set point” and a gradually declining CD4 cell count in the absence of HAART.

The HIV viral load set point was modeled as a constant value during the asymptomatic phase, but the set point may vary among individuals. The HIV log_10_ viral load set point was chosen randomly from a normal distribution (mean 4.5 log_10_ copies/ml, range 4.0-5.0 log_10_ copies/ml).[6]

The rate of decline in CD4 cell count in a particular quarter is greater at higher HIV viral loads. We used estimates of the rate of CD4 decline for different ranges of HIV viral load reported by Rodriguez et al.[11] These estimates were based on analyses of the Research in Access to Care for the Homeless (REACH) cohort and the San Francisco Men's Health Study (SFMHS) cohort data (Table 1). The rate of CD4 decline for the set point viral load of 4.5 log_10_ copies/ml is 14.1 cells/µL/quarter. The maximum rate of decline (19.5 cells/µL/quarter) occurs when the viral load exceeds 4.6 log_10_ copies/ml, while the minimum decline (5.1 cells/µL/quarter) occurs for a viral load less than 2.7 log_10_ copies/ml.

**Symptomatic HIV Infection/AIDS**

In the PATH model, individuals transition into the symptomatic HIV infection or AIDS phase of the model if they develop symptoms of an opportunistic illness (OI) or if their CD4 cell count drops below 200 cells/µL.[12] Individuals were assumed to remain in the symptomatic phase until their OI cleared (in one quarter) or their CD4 cell count rebounded after administration of HAART. Notable differences in symptomatic and asymptomatic HIV infection included higher costs and lower utilities (discussed in later sections of this appendix) during an episode of OI. Persons with a history of OI or AIDS were also assumed to have higher inpatient and outpatient medical services costs than those without such a history (discussed in later sections of this appendix).

We assumed that individuals could acquire only a single OI per quarter, but could have an OI in any number of different quarters. The probability of having an OI increased with a decline in CD4 cell count (Table 1). We modeled six categories of such illnesses: *Pneumocystis jiroveci* pneumonia (PCP), *Mycobacterium avium* complex, Toxoplasmosis, Cytomegalovirus infection, fungal infections, and other OIs. The cumulative probability, which represents the quarterly transition probability of moving from asymptomatic HIV infection to symptomatic HIV infection or AIDS due to an OI, is 2.2% at a CD4 cell count of 201-300 cells/µL compared to 0.3% at CD4 cell counts higher than 500 cells/µL.[13,14]

Transition rates are generally expressed in units of person-time, and probabilities, in monthly or yearly units. Because a quarter is our time unit of progression, we converted all rates or other probabilities to quarterly probabilities. To do so, we assumed that the transition rates were constant over time and that the probabilities could be defined by an exponential distribution. For example, to convert from a monthly probability to a quarterly probability, we first estimated a corresponding annual rate, and then converted the annual rate to a quarterly probability. The relationship between the probability of transitioning (p), annual rate (µ), and unit of time (t) is given by the formula [15]: p(µ, t) = 1- e^-µt^ . Consider converting the monthly probability of an OI = 0.037 to a quarterly probability. We first estimate the annual rate, µ = - (1/t) * ln (1 - p). Substituting t = 1/12, and p = 0.037, we get an annual rate = 0.452 OIs per year. To estimate the quarterly probability, substitute μ = 0.452 and t= (1/4) in the formula, p= 1- e^-µ^*^t^*, to get a quarterly probability of 0.106.

**Mortality Among Individuals Not on HAART**

HIV-infected individuals in the model can die from causes either related to HIV/AIDS or other factors. For individuals who are not yet on treatment, we used different quarterly probabilities of death for HAART-naïve individuals infected through sexual transmission [16] or through IDU transmission.[17] These quarterly probabilities of death, which represent the combined HIV/AIDS-related and non-HIV/AIDS-related mortality, increase as the CD4 cell count declines. We assumed that the probability of death is constant throughout a specific range of CD4 cell counts as depicted in Table 1.

The maximum number of years of life remaining for an individual infected with HIV in the PATH model is limited by life expectancy at the age of infection based on the 2004 United States life tables. [18] For example, individuals infected at 35 years of age can live for a maximum of 44.5 additional years—their average life expectancy as predicted by the life tables. [18]

**Diagnosis of HIV Infection and Setting**

In this model, an HIV-infected individual can be diagnosed only if tested in a specific setting. Input parameters that define a setting include the CD4 cell count at diagnosis, the undiagnosed HIV seropositivity rate of individuals who are tested in that setting, and the program costs associated with identifying an HIV-infected individual in that setting. We assumed that an individual was diagnosed with HIV at a specific CD4 cell count, based on the distribution of CD4 cell counts at diagnosis in the settings under consideration. A detailed description of test settings is provided in the main manuscript that discusses the application of the PATH model.

We assumed that HIV testing is conducted using either a conventional enzyme-linked immunosorbent assay (EIA) or a rapid test [19] depending upon the specific setting. All positive EIA and rapid tests were assumed to be followed up with a confirmatory Western blot.

**Linkage to Care**

Our assumptions about linkage to care also varied by diagnostic setting. We assumed that persons diagnosed as inpatients were linked to care in the quarter following diagnosis. For persons diagnosed in all other settings, we assumed that 65% were linked to care in the quarter after diagnosis. Another 15% were assumed to be linked to care by the time their CD4 count decreased to 200 cells/µL, and the remaining 20% were assumed to be diagnosed as inpatients and linked to care when their CD4 count decreased to 36 cells/µL, the median CD4 count at diagnosis in hospital inpatient settings.[20] These assumptions are consistent with data from studies of linkage to care in various settings.[21-24]

**Modeling HAART Regimens**

In the PATH model base-case, individuals diagnosed with HIV and linked to care become eligible for HAART when their CD4 cell count is at or below 350 cells/µL.[25] [26] Thus, persons diagnosed at higher CD4 cell counts have to wait until their CD4 cell count drops to 350 cells/µL, whereas those diagnosed at lower CD4 cell counts initiate HAART in the next quarter. A policy of starting HAART at a CD4 cell count of 500 cells/µL was also analyzed.[25]

Individuals on HAART follow a different disease progression compared with the natural history of HIV. In general, HAART is associated with suppressed HIV viral load, higher CD4 cell counts, improved life expectancy, improved quality of life, and higher costs. [27,28] We modeled a maximum of four treatment regimens followed by one non-suppressive, or salvage regimen.[26] The schematic flowchart for suppressive and non-suppressive HAART is provided in Figure 2.

The convenient dosing, reduced toxicity and improved tolerability of newer HAART drugs has resulted in an improved durability of regimens. [29] Recent studies have shown that early initiation of HAART may improve health outcomes. [30], [31] We incorporated the emerging evidence for better adherence, reduced toxicity, improved tolerability, and the benefits of early initiation of HAART through a variety of assumptions. First, we assumed that all four regimens had an 80% probability of successfully suppressing HIV viral load. (Expert opinion, 2009) Second, we assumed that individuals with early access to HAART had a better chance of survival [28], [27] and an ability to attain higher CD4 cell counts [32], [33] compared to those who accessed HAART later in their disease progression.

Once suppressed, HIV viral load was assumed to remain constant at 1.3 log_10_ copies/ml (range 1.0 - 2.7 log_10_ copies/ml) [34] as long as the regimen was effective. When a particular treatment regimen ceased to be effective, we assumed that the HIV viral load rebounded to 3.7 log_10_ copies/ml (range 3.1 – 4.5 log_10_ copies/ml). [35] The individual still remained on the same regimen and incurred the treatment costs for one additional quarter because HIV viral load rebound was likely not apparent until then. At the end of this quarter, the increased HIV viral load became evident in the new CD4 cell count and HIV viral load tests, and the patient and provider decided upon a further course of treatment. We assumed that the individual was started on the next successive therapy if all four regimens had not been exhausted. When a subsequent therapy was effective, the HIV viral load was again suppressed to 1.3 log_10_ copies/ml (range 1.0 - 2.7 log_10_ copies/ml). [34] When a subsequent therapy ceased to be effective, we again assumed that HIV viral load rebounded to 3.7 log_10_ copies/ml (range 3.1 – 4.5 log_10_ copies/ml). [35]

We used a geometric distribution to model the duration that each regimen was effective. The distribution was based on the assumption that 7.4% of HIV-infected patients experienced a viral load rebound one year after starting the first HAART regimen. [7], [35] This rebound percentage one year after HAART increased by 18% for each subsequent regimen to 8.7%, 10.3%, and 12.2% for the second through fourth regimens.[36]

When a regimen was not effective, we assumed that CD4 cell counts declined at the same rate as they would during the natural history of HIV. [11] When a regimen was effective and HIV viral loads were suppressed, we assumed that CD4 cell counts increased. The maximum CD4 cell count that could be achieved during sustained HIV viral load suppression depended upon the CD4 cell count at initiation of the first HAART regimen. We used median estimates from Gras et al. [32] to model different maximum CD4 cell counts that could be reached after initiation of HAART. The estimates were based on an analysis of patients from the AIDS Therapy Evaluation Project, Netherlands (ATHENA) national observational HIV cohort study. According to this study, an individual who had extended HIV viral load suppression following initiation of HAART at a CD4 cell count of 50 cells/µL could attain a CD4 cell count of up to 410 cells/µL, whereas an individual who started HAART at a CD4 cell count of over 500 cells/µL could attain a CD4 cell count of up to 870 cells/µL (Table 1).

We assumed that the rate of increase in CD4 cell count was highest during the first two quarters after initiation of HAART (68 cells/µL/quarter), then decreased to 40 cells/µL/quarter from the third to the twelfth quarter, and to zero cells/µL/quarter thereafter. [32] Once the CD4 cell counts reached the maximum levels discussed above, we assumed they stabilized and there were no further increases in CD4 cell counts even if the HIV viral load remained suppressed.

After all suppressive regimens are exhausted, the individual is put on salvage or non-suppressive therapy. The model no longer allows HIV viral load suppression to undetectable levels whether or not the individual is treated with a salvage regimen. We assumed that the HIV viral load increased by 0.8 log_10_ copies/ml (range 0 to 1.5 log copies/ml)[37] above the HIV viral load set point value during salvage regimen and by 1 log_10_ copies/ml (range 0 - 2.0 log copies/ml)[37] above the set point after the onset of AIDS. We modeled for the CD4 cell count to decline in a manner similar to that described for natural disease progression.[11]

The ART Cohort Collaboration published mortality risks that depend on the range of CD4 cell counts at initiation of HAART, age, mode of transmission (sexual or IDU), and whether patients had symptoms of an AIDS-defining OI. We used the ART Cohort Collaboration’s [28], [27] five-year probability of death to estimate the quarterly probability of death in the model based on the values of these variables at the time the patient initiated the first HAART regimen (Table 1).

**QALYs Lost to HIV Infection**

For each quarter that individuals survive in the model, we assigned quality-of-life weights ranging from 0 (death) to 1 (perfect health) based on the individual’s CD4 cell count or the presence of an OI during that quarter. We used quality-of-life utility weights from the meta-analysis conducted by Tengs and Lin.[38] QALYs were assigned for each quarter and discounted at 3% per year.[39] Once we aggregated QALYs over the life of an individual, we then subtracted the sum from the QALYs associated with an HIV-uninfected person, assuming a QALY of 1 (good health) for the entire life expectancy at the age of infection, [18] and estimated the QALYs lost due to HIV infection. Measuring QALYs lost due to infection results in consistent quality of life estimates when transmissions to partners are included in the model (discussed below). A decrease in QALYs lost in one setting compared with another represents a gain in QALYs in the first setting.

**Costs**

We estimated costs from the provider perspective and did not include costs incurred by the patient, such as out-of-pocket expenses and productivity losses. Quarterly costs, based on disease treatment costs estimated by Schackman et al.[40], included inpatient and outpatient medical services and the costs of HAART. We included the costs for tests for CD4 cell count and HIV viral load in each quarter following HIV diagnosis and an HIV genotype test before the beginning of each HAART regimen.

We also added costs for treating OIs when they occurred and an additional cost during the patient’s last month of life. The OI costs varied by the type of illness. The costs incurred during the last month of life included the costs of terminal hospitalization and varied by whether an individual had an OI during that time.[40] Patients with a history of an OI or AIDS had higher inpatient and outpatient medical costs than those without this history. Details of these costs are provided in Table 1.

We assumed that persons with unrecognized HIV infection had the same inpatient and outpatient medical services utilization costs as those for persons who were diagnosed with HIV but had not started HAART. Undiagnosed persons were not assigned any other associated costs. However, we added one-time diagnostic costs in the quarter during which an individual was diagnosed. These costs, which vary among the settings in which individuals are diagnosed, are discussed in detail in the main paper.

All costs were adjusted to 2009 US dollars using the U.S. city average medical care consumer price index. We assumed there were no changes in technology that would affect real costs over the analytic timeframe, and we discounted costs at a rate of 3% per year. [39]

**Transmission**

We estimated a quarterly probability of HIV transmission per infected individual to predict if a transmission occurred in a given quarter. We assumed that infection in the first quarter represented the acute phase of HIV infection that could not be diagnosed with EIA tests. Persons with acute infection remain unaware of their status in this model, have higher HIV viral loads, [4], [5] and are more infectious. All persons unaware of their infection may continue to engage in high-risk behavior that increases HIV transmission.[41] Persons on HAART have reduced HIV viral loads and, hence, reduced chance of transmission.[25]

We derived a quarterly probability of transmission from an annual transmission rate, defined as the average number of HIV transmissions per person living with HIV/AIDS, for those acutely infected and unaware of their infection (N_1_), those non-acutely infected and unaware (N_2_), and for those non-acutely infected who are aware and either not on (N_3_) or on (N_4_) a HAART regimen. These population-based average rates per person were derived from surveillance data on HIV incidence and prevalence and the proportion of people who are unaware of their HIV infection.

Of the 1,106,300 persons currently estimated to be living with HIV, 232,700 are estimated to be unaware of their diagnosis.[42] We estimated that 7,558 of these persons are acutely infected and unaware (N_1_), based on an estimate of 56,300 new annual infections[1] and assuming an average duration of acute infection of 49 days.[5] The remaining 225,142 persons are non-acutely infected and unaware (N_2_). Of the 873,600 individuals who are aware of their infection, we estimated that 419,328 are not on HAART (N_3_) and 454,272 are on HAART (N_4_), given the assumptions that 65% of the non-acute aware are linked to care and 80% of those individuals are on HAART.

Following Pinkerton[5] and Prabhu et al.[43], we defined γ_k_ as the transmission rate in group N_k_ and

I = total number of transmissions = γ_1_N_1_ + γ_2_N_2_ + γ_3_N_3_ + γ_4_N_4_ (1)

The proportion of transmissions attributed to a particular group is γ_k_N_k_ / I. For example, the proportion of transmissions due to the acute phase is

I_A_ = γ_1_N_1_ / [γ_1_N_1_ + γ_2_N_2_ + γ_3_N_3_ + γ_4_N_4_] (2)

Define the following transmission rate ratios:

µ_12_ = γ_1_ / γ_2_ = acute unaware / non-acute unaware

µ_23_ = γ_2_ / γ_3_ = non-acute unaware / non-acute aware, not on HAART

µ_43_ = γ_4_ / γ_3_ = non-acute aware, on HAART / non-acute aware, not on HAART

(µ_12_) (µ_23_) = γ_1_ / γ_3_ = acute unaware / non-acute aware, not on HAART

Dividing both the numerator and the denominator of equation (2) by γ_3_ gives,

I_A_ = (µ_12_) (µ_23_) N_1_ / [(µ_12_) (µ_23_) N_1_ + µ_23_ N_2_ + N_3_ + µ_43_ N_4_] (3)

The values for the transmission rate ratios are as follows:

µ_12_ = 8.10

µ_23_ = 2.29

µ_43_ = 0.20

The value of 8.10 for µ_12_ (acute unaware / non-acute unaware) was derived by Pinkerton[5] as an average of an upper-bound value of 12.0, based on a behavioral model of men who have sex with men (MSM) by Rapatski et al.[44], and a lower-bound value of 4.2, derived from a study by Pilcher et al.[45] of male-to-female transmission. We derived the numerators and denominators of the transmission rate ratios, µ_23_ (non-acute unaware/ non-acute aware not on HAART) and µ_43_ (non-acute aware on HAART / non-acute aware not on HAART), by dividing the % of overall transmissions associated with each group by the % of total persons living with HIV in each group.

We estimated that 51% of all transmissions are attributed to the 21% of persons living with HIV who are unaware of their status.[42] The 51% transmission estimate for persons unaware was derived from the analysis by Marks et al.[41] of the differences in transmission rates among those aware and unaware of their serostatus. Our analysis was based on the underlying assumption that 65% of those aware of their serostatus are linked to care, of whom 80% are on HAART, and, of those on HAART, 80% have a suppressed viral load. Thus, we modified the Marks et al. Rvl parameter, the proportion of HIV-infected aware persons who potentially transmit to at-risk partners (1 – [the proportion of aware HIV-infected individuals who are linked to care and on HAART times the proportion of those who are aware and linked to care that have very low viral load]) , from 0.67 to 0.584.

Non-acute aware individuals not on HAART include those aware individuals who are not linked to care and those who are linked to care but have not yet initiated HAART. We estimated that this group represents 38% of those living with HIV and that they account for 40% of all transmissions. Therefore, µ_23_ = (0.51 / 0.21) / (0.40 / 0.38) = 2.29. Those who are aware and have initiated HAART account for 41% of persons living with HIV and for 9% of the transmissions. Therefore, µ_43_ = (0.09 / 0.41) / (0.40 / 0.38) = 0.20.

Substituting these values into equation (3) gives a value of I_A_ equal to 0.12. This value was then multiplied by the 47,207 annual estimated infections acquired through sexual transmission[1] to derive an estimated 5,673 annual infections from the acutely infected unaware. Dividing this number by the estimated number of persons acutely infected (N_1_ = 7,558) gives an annual transmission rate for the acutely infected of 0.751. Annual transmission rates for the other groups of 0.093 for N_2_ (non-acute unaware), 0.041 for N_3_ (non-acute aware not on HAART), and 0.008 for N_4_ (non-acute aware on HAART) were derived in a similar manner. Quarterly transmission probabilities were derived from these annual rates. We estimated different rates for IDU transmission for persons with acute infection, undiagnosed non-acute infection and diagnosed non-acute infection using Sanders et al [37] and Zaric et al. [46]

The individuals initially entering the PATH model are considered the first generation of HIV-infected persons or index patients. Transmissions from index patients result in a second generation of HIV-infected persons. We then tracked disease progression, along with costs incurred and QALYs lost of this second generation of HIV-infected partners, and added the costs incurred and QALYs lost for the first and second generation HIV-infected persons to predict the total costs incurred and QALYs lost from HIV infection when transmission is included in the model. All costs and QALYs for partners were discounted from the date of infection of the index person.

Because the analysis was designed to investigate the cost-effectiveness of strategies for testing index patients for HIV in various settings, disease progression and treatment assumptions for infected partners were held constant for all partners. We assumed that partners infected through either sexual transmission or injection drug use would be diagnosed at a CD4 cell count of 500 cells/µL, and that they would be linked to care based on assumptions similar to those for index persons in EDs and STD clinics. This assumption was necessary because we did not want differences in treatments received by partners to influence the cost-effectiveness analysis.

**Limitations**

This is a model-based analysis that uses parameter assumptions derived from recent published literature. The model does not explicitly address the issue of immunological non-responders [47], [48], i.e., patients who are receiving HAART, but who do not exhibit a marked increase in their CD4 cell count. The model does not separately study the effect of co-morbidities associated with HIV, although the mortality rates include deaths related and unrelated to HIV/AIDS, and the costs include all health care utilization by those with HIV. We did not explicitly model resistance to HAART or specific adverse reactions to HAART. However, these factors were included in our measures of the effectiveness of the HAART regimens.

Our individual sampling model considers transmission to partners of the index patients in only a limited way. In our analysis we used previously published transmission rate estimates to project transmission to a first generation of partners, thus underestimating cost-effectiveness by not including transmission from infected partners to subsequent rounds of partners. However, we developed and used a complex set of transmission rates that included acute and non-acute infection, awareness of infection, and the impact of the HAART regimens. The strength of our model lies in its detailed projection of disease progression and its accounting for the costs and QALYs associated with different disease stages.

**Table 1, Complete List of Input Parameters**

| **Variable** | **Base Case Value** | **Range** | **Source** |
| --- | --- | --- | --- |
| Age at infection (years) | 35 | 30 - 40 | 1 |
| Discount rate for costs and quality-adjusted life years (QALYs) | 3% |  | [39] |
| **Natural Disease Progression** |  |  |  |
| CD4 cell count when infected (cells/µL) | 900 | 750 - 900 | [10] |
| Acute phase HIV viral load (log_10_ copies/ml) | 5.3 | 4.4 – 6.2 | [8,9] |
| HIV viral load set point (log_10_ copies/ml) | 4.5 | 4.0 – 5.0 | [6,7] |
| Natural rate of CD4 cell count decline (cells/µL/quarter) as a function of HIV viral load stratum (log_10_ copies/ml) |  |  | [11] |
| ≤ 2.7 | 5.1 | 2.4 – 7.8 |  |
| 2.7 – 3.3 | 9.9 | 7.2 – 12.3 |  |
| 3.3 – 4.0 | 12.0 | 9.9 – 13.8 |  |
| 4.0 – 4.6 | 14.1 | 11.7 – 16.2 |  |
| ≥ 4.6 | 19.5 | 17.1 – 21.9 |  |
| **Quarterly Probability of Developing an Opportunistic Infection (OI) (%)** |  |  | [13,14] |
| *Pneumocystis pneumonia* (PCP) | 0.1 – 10.7^2^ |  |  |
| *Mycobacterium avium* complex | 0.0 – 3.6 |  |  |
| Toxoplasmosis | 0.0 – 0.8 |  |  |
| Cytomegalovirus infection | 0.0 – 5.5 |  |  |
| Fungal infection | 0.0 – 3.3 |  |  |
| Other | 0.1 – 11.4 |  |  |
| Cumulative probability for all OIs | 0.3 – 35.3 |  |  |
| **Quarterly Probability of Death for Highly Active Antiretroviral Therapy (HAART)-Naïve Individuals (%)** |  |  |  |
| Sexual transmission: CD4 cell count (cells/µL) |  |  | [16] |
| ≥ 650 | 0.043 |  |  |
| 500 – 649 | 0.050 |  |  |
| 350 – 499 | 0.080 |  |  |
| 200 – 349 | 0.145 |  |  |
| 50 – 199 | 0.767 |  |  |
| < 50 | 4.900 |  |  |
| Injection drug use (IDU) transmission: CD4 cell count (cells/µL) |  |  | [17] |
| ≥ 350 | 1.069 |  |  |
| 200 – 349 | 1.486 |  |  |
| < 200 | 4.068 |  |  |
| **HAART Regimens** |  |  |  |
| Minimum CD4 cell count to initiate HAART (cells/µL) | 350 / 500 |  | [25] |
| Suppressed HIV viral load level (log_10_ copies/ml) | 1.3 | 1.0 – 2.7 | [34] |
| Rebound HIV viral load level (log_10_ copies/ml) | 3.7 | 3.1 – 4.5 | [35] |
| Maximum number of HAART regimens | 4 |  | 3 |
| Probability of virologic suppression in HAART regimens 1 – 4 | 0.80 |  | 3 |
| Rate of HIV viral load rebound (% experiencing rebound after one year, first regimen) | 7.4 |  | [7,35] |
| Rate of HIV viral load rebound for each successive regimen | 1.18 |  | [36] |
| HIV viral load above set-point during salvage therapy (log_10_ copies/ml) | 0.8 | 0.0 – 1.5 | [37] |
| HIV viral load above set-point during salvage therapy after onset of AIDS (log_10_ copies/ml) | 1.0 | 0.0 – 2.0 | [37] |
| Quarterly increase in CD4 cell count during HIV viral load suppression (cells/µL/quarter) |  |  | [32] |
| Quarters 1 – 2 | 68 |  |  |
| Quarters 3 – 12 | 40 |  |  |
| Quarters 12+ | 0 |  |  |
| Maximum CD4 cell count achieved based on CD4 cell count at initiation of HAART (cells/µL) |  |  | [32] |
| < 50 | 410 |  |  |
| 50 – 200 | 548 |  |  |
| 201 – 350 | 660 |  |  |
| 351 - 500 | 780 |  |  |
| > 500 | 870 |  |  |
| **Quarterly Probability of Death After Initiation of HAART (%)** |  |  | [27,28] |
| Sexual transmission |  |  |  |
| No AIDS symptoms |  |  |  |
| Age 16 – 29 years | 0.09 – 0.26^4^ |  |  |
| Age 30 – 39 years | 0.12 – 0.32 |  |  |
| Age 40 – 49 years | 0.15 – 0.43 |  |  |
| Age ≥ 50 years | 0.29 – 0.81 |  |  |
| Clinical symptoms of AIDS |  |  |  |
| Age 16 – 29 years | 0.19 – 0.53 |  |  |
| Age 30 – 39 years | 0.25 – 0.69 |  |  |
| Age 40 – 49 years | 0.32 – 0.93 |  |  |
| Age ≥ 50 years | 0.64 – 1.77 |  |  |
| Injection drug use transmission |  |  |  |
| No AIDS symptoms |  |  |  |
| Age 16 – 29 years | 0.27 – 0.75 |  |  |
| Age 30 – 39 years | 0.35 – 0.99 |  |  |
| Age 40 – 49 years | 0.46 – 1.30 |  |  |
| Age ≥ 50 years | 0.87 – 2.44 |  |  |
| Clinical symptoms of AIDS |  |  |  |
| Age 16 – 29 years | 0.58 – 1.63 |  |  |
| Age 30 – 39 years | 0.75 – 2.06 |  |  |
| Age 40 – 49 years | 0.99 – 2.77 |  |  |
| Age ≥ 50 years | 1.84 – 5.11 |  |  |
| **Utility Weights to Estimate Quality Adjusted Life Years (QALYs)** |  |  | [38] |
| OI or CD4 count < 200 cells/µL | 0.702 |  |  |
| CD4 cell count ≥ 200, < 350 cells/µL | 0.818 |  |  |
| CD4 cell count > 350 cells/µL | 0.935 |  |  |
| **Quarterly Costs (2009 $)** |  |  | [40] |
| Inpatient and outpatient resource utilization |  |  |  |
| Without history of AIDS-defining OI, on HAART | 905 – 2,929^5^ |  |  |
| Without history of AIDS-defining OI, not on HAART | 1,097 – 6,007^5^ |  |  |
| With history of AIDS-defining OI, on HAART | 1,984 |  |  |
| With history of AIDS-defining OI, not on HAART | 2,071 |  |  |
| Additional costs of opportunistic infections (each occurrence) | 3,492 – 20,542^6^ |  |  |
| Additional costs of last month of life without OI in the last month | 24,671 |  |  |
| Additional costs of last month of life with OI in the last month | 35,750 |  |  |
| Additional cost of HAART (each quarter) | 4,143 – 13,699^7^ |  |  |
| CD4 cell count test (one each quarter) | 80 |  |  |
| HIV viral load test (one each quarter) | 144 |  |  |
| HIV genotype test (beginning of each regimen) | 436 |  |  |
| **Annual Rates of Transmission (# events per year per person)** |  |  |  |
| Sexual transmission |  |  | Derived from [5,43] |
| Acute | 0.751 |  |  |
| Non-acute unaware | 0.093 |  |  |
| Non-acute aware, not on HAART | 0.041 |  |  |
| Non-acute aware, on HAART | 0.008 |  |  |
| IDU transmission |  |  | Derived from [5,37,43,46] |
| Acute | 1.336 |  |  |
| Non-acute unaware | 0.165 |  |  |
| Non-acute aware, not on HAART | 0.072 |  |  |
| Non-acute aware, on HAART | 0.014 |  |  |

^1^Writen communication, R. Song, Centers for Disease Control and Prevention, June, 2008.

^2^The lower and upper bounds for various types of OIs reflect probabilities for CD4 cell counts of > 500 cells/µL and 0 – 50 cells/µL respectively. Probabilities of an OI at intermediate CD4 cell counts lie within these bounds.

^3^Expert opinion (2009).

^4^The lower and upper bounds reflect the probability of death for CD4 cell counts ≥ 350 cells/µL and < 25 cells/µL, respectively. Probabilities of death at intermediate CD4 cell counts lie within these bounds.

^5^The lower and upper bounds for costs reflect costs for CD4 cell counts > 500 cells/µL and ≤ 50 cells/µL, respectively. Costs for intermediate CD4 cell counts lie within these bounds.

^6^These numbers represent costs for different opportunistic illnesses.

^7^The lower and upper bounds for costs reflect costs for the first and fourth HAART regimens. Costs for the other regimens lie in between these values.

**Figure 1: Schematic flow chart (PATH model overview)**


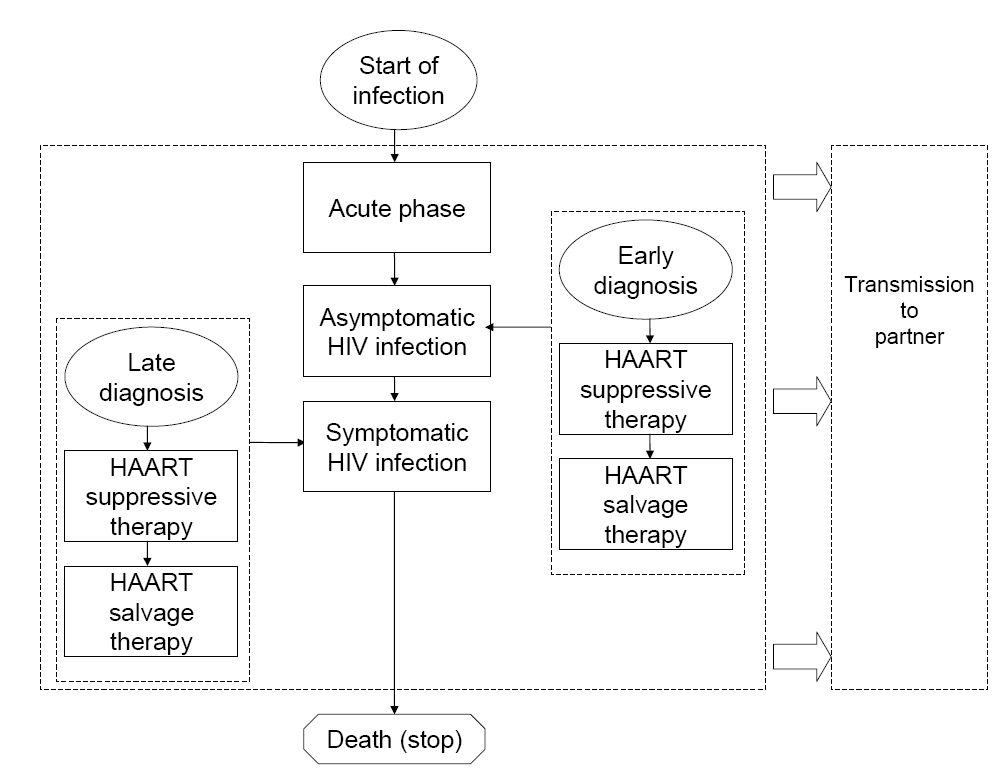


**Figure 2: Schematic Flowchart for Highly Active Antiretroviral Therapy (HAART) regimens**

*therapy*

*successful*

therapy

fails

*rebound*

*therapy*

*successful*

therapy

fails

*rebound*

*therapy*

*successful*

therapy

fails

*rebound*

*therapy*

*successful*

therapy

fails

*rebound*

Patient stays on

regimen 4 until viral

load rebounds

Patient stays on

regimen 3 until viral

load rebounds

Patient stays on

regimen 2 until viral

load rebounds

Patient stays on

regimen 1 until viral

load rebounds

Death

Start

HAART

Start Suppressive

therapy: regimen 1

Start Suppressive

therapy: regimen 2

Start Suppressive

therapy: regimen 3

Start salvage therapy

Start Suppressive

therapy: regimen 4

**References**

1. Hall HI, Song R, Rhodes P, Prejean J, An Q, et al. (2008) Estimation of HIV Incidence in the United States. JAMA 300: 520-529.

2. Quinn TC (1997) Acute primary HIV infection. JAMA 278: 58-62.

3. Lindback S, Karlssond AC, Mittlergh J, Blaxhultb A, Carlssone M, et al. (2000) Viral dynamics in primary HIV-1 infection. AIDS 14: 2283-2291.

4. Pilcher CD, Eron Jr. JJ, Galvin S, Gay C, Cohen MS (2004) Acute HIV revisited: new opportunities for treatment and prevention. The Journal of Clinical Investigation 113: 937-945.

5. Pinkerton SD (2007) How many sexually-acquired HIV infections in the USA are due to acute-phase HIV transmission? AIDS 21: 1625-1629.

6. Herbeck JT, Gottlieb GS, Li X, Hu Z, Detels R, et al. (2008) Lack of Evidence for Changing Virulence of HIV-1 in North America. PLOS One 2.

7. Vo TTN, Ledergerber B, Keiser O, Hirschel B, Furrer H, et al. (2008) Durability and Outcome of Initial Antiretroviral Treatments Received during 2000-2005 by Patients in the Swiss HIV Cohort Study. JID 197: 1685-1694.

8. Schacker TW, Collier AC, Hughes JP, Shea T, Corey L (1996) Clinical and epidemiologic features of primary HIV infection. Ann Intern Med 125: 257-264 (erratum: Ann Intern Med 1997;1126:1174).

9. Schacker TW, Hughes JP, Shea T, Coombs RW, Corey L (1998) Biological and Virologic Characteristics of Primary HIV Infection. Ann Intern Med 128: 613-620.

10. Turner BJ, Hecht FM, Ismail RB (1994) CD4+ T-lymphocyte measures in the treatment of individuals infected with human immuno deficiency virus type 1: a review for clinical practitioners. Arch Intern Med 154: 1561-1573.

11. Rodríguez B, Sethi AK, Cheruvu VK, Mackay W, Bosch RJ, et al. (2006) Predictive value of plasma HIV RNA level on rate of CD4 t-cell decline in untreated HIV infection. JAMA 296: 1498-1506.

12. Centers for Disease Control and Prevention (1992) 1993 revised classification system for HIV infection and expanded surveillance case definition for AIDS among adolescents and adults MMWR Recommendations and Reports 18: 1-19.

13. Paltiel AD, Weinstein MC, Kimmel AD, Seage III GR, Losina E, et al. (2005) Expanded Screening for HIV in the United States — An Analysis of Cost-Effectiveness. New England Journal of Medicine 352: 586-595.

14. Weinstein MC, Goldie SJ, Losina E, Cohen CJ, Baxter JD, et al. (2001) Use of Genotypic Resistance Testing To Guide HIV Therapy: Clinical Impact and Cost-Effectiveness. Ann Intern Med 134: 440-450.

15. Miller DK, Homan SM (1994) Determining transition probabilities: confusion and suggestions. Medical decision making 14: 52-58.

16. The United Kingdom Collaborative HIV Cohort (CHIC) Study (2007) Rate of AIDS diseases or death in HIV-infected antiretroviral therapy-naive individuals with high CD4 cell count. AIDS 21: 1717-1721.

17. Wang C, Vlahov D, Galai N, Bareta J, Strathdee SA, et al. (2004) Mortality in HIV-Seropositive versus -Seronegative Persons in the Era of Highly Active Antiretroviral Therapy: Implications for When to Initiate Therapy. The Journal of Infectious Diseases 190: 1046-1054.

18. Arias E (2007) United States Life Tables, 2004. National Vital Statistics Report 56: 1-40.

19. Greenwald JL, Burstein GR, Pincus J, Branson B (2006) A Rapid Review of Rapid HIV Antibody Tests. Current Infectious Disease Reports 8: 125-131.

20. Jean-Jacques M, Walensky RP, Aaronson WH, Chang Y, Freedberg KA (2008) Late diagnosis of HIV infection at two academic medical centers: 1994-2004. AIDS Care 20: 8,977-983.

21. Torian LV, Wiewel EW, Liu K-L, Sackoff JE, Frieden TR (2008) Risk Factors for Delayed Initiation of Medical Care After Diagnosis of Human Immunodeficiency Virus. Arch Intern Med 168: 1181-1187.

22. Zetola N, Bernstein K, Ahrens K, Marcus J, Philip S, et al. (2009) Using surveillance data to monitor entry into care of newly diagnosed HIV-infected persons: San Francisco, 2006-2007. BMC Public Health 9: 17.

23. Reed JB, Hanson D, McNaghten AD, Bertolli J, Teshale E, et al. (2009) HIV Testing Factors Associated with Delayed Entry into HIV Medical Care among HIV-Infected Persons from Eighteen States, United States, 2000-2004. AIDS Patient Care and STDs 23: 765-773.

24. Marks G, Gardner LI, Craw J, Crepaz N (2010) Entry and Retention in Medical Care Among HIV-Diagnosed Persons: A Meta-Analysis. AIDS 24.

25. Panel on Antiretroviral Guidelines for Adults and Adolescents (December 1, 2009) Guidelines for the use of antiretroviral agents in HIV-1-infected adults and adolescents. In: Department of Health and Human Services, editor. pp. 1-161.

26. Dybul M, Fauci AS, Bartlett JG, Kaplan JE, Pau AK (2002) Guidelines for using antiretroviral agents among HIV-infected adults and adolescents. Ann Intern Med 137: 381-433.

27. Antiretroviral Therapy Cohort Collaboration (2007) Importance of baseline prognostic factors with increasing time since initiation of highly active antiretroviral therapy: collaborative analysis of cohorts of HIV-1-infected patients. Journal of acquired immune deficiency syndromes 46: 607-615.

28. Antiretroviral Therapy Cohort Collaboration (2007) Prognosis of HIV-1-infected patients up to 5 years after initiation of HAART: collaborative analysis of prospective studies. AIDS 21: 1185-1197.

29. Willig JH, Abroms S, Westfall AO, Routman J, Adusumilli S, et al. (2008) Increased regimen durability in the era of once-daily fixed-dose combination antiretroviral therapy. AIDS 22: 1951-1960.

30. Kitahata MM, Gange SJ, Abraham AG, Merriman B, Saag MS, et al. (2009) Effect of Early versus Deferred Antiretroviral Therapy for HIV on Survival. The New England Journal of Medicine 306: 1815-1826.

31. When To Start Consortium (2009) Timing of initiation of antiretroviral therapy in AIDS-free HIV-1-infected patients: a collaborative analysis of 18 HIV cohort studies. Lancet 2009: 1352-1363.

32. Gras L, Kesselring AM, Griffin JT, van Sighem AI, Fraser C, et al. (2007) CD4 Cell Counts of 800 Cells/mm3 or Greater After 7 Years of Highly Active Antiretroviral Therapy Are Feasible in Most Patients Starting With 350 Cells/mm3 or Greater. Journal of acquired immune deficiency syndromes 45: 183-192.

33. Moore RD, Keruly JC (2007) CD4+ Cell Count 6 Years after Commencement of Highly Active Antiretroviral Therapy in Persons with Sustained Virologic Suppression. Clinical Infectious Diseases 44: 441-446.

34. Raboud JM, Montaner JSG, Conway B, Rae S, Reiss P, et al. (1998) Suppression of plasma viral load below 20 copies/ml is required to achieve a long-term response to therapy. AIDS 12: 1619-1624.

35. The United Kingdom Collaborative HIV Cohort (CHIC) Study (2008) Treatment switches after viral rebound in HIV-infected adults starting antiretroviral therapy: multicentre cohort study. AIDS 22: 1943-1950.

36. Smith CJ, Phillips AN, Dauer B, Johnson MA, Lampe FC, et al. (2009) Factors associated with viral rebound among highly treatment-experienced HIV-positive patients who have achieved viral suppression. HIV Medicine 10: 19-27.

37. Sanders GD, Bayoumi AM, Sundaram V, Bilir SP, Neukermans CP, et al. (2005) Cost-Effectiveness of Screening for HIV in the Era of Highly Active Antiretroviral Therapy. New England Journal of Medicine 352: 570-585.

38. Tengs TO, Lin TH (2002) A meta-analysis of utility estimates for HIV/AIDS. Medical decision making 22: 475-481.

39. Gold MR, Siegel JE, Russell LB, Weinstein MC, editors (1996) Cost-Effectiveness in Health and Medicine. New York: Oxford University Press.

40. Schackman BR, Gebo KA, Walensky RP, Losina E, Muccio T, et al. (2006) The lifetime cost of current Human Immunodeficiency Virus care in the United States. Medical Care 44: 990-997.

41. Marks G, Crepaz N, Janssen RS (2006) Estimating sexual transmission of HIV from persons aware and unaware that they are infected with the virus in the USA. AIDS 20: 1447-1450.

42. Centers for Disease Control and Prevention (2008) HIV Prevalence Estimates -- United States, 2006. Morbidity and Mortality Weekly Report 57: 1073-1076.

43. Prabhu VS, Hutchinson AB, Farnham PG, Sansom SL (2009) Sexually-acquired HIV infections in the USA due to acute-phase HIV transmission: An update. AIDS 23: 1792-1794.

44. Rapatski BL, Suppe F, Yorke JA (2005) HIV Epidemics Driven by Late Disease Stage Transmission. Journal of Acquired Immune Deficiency Syndromes 38: 241-253.

45. Pilcher Christopher D, Tien Hsiao C, Eron Joseph J, Jr., Vernazza Pietro L, Leu S-Y, et al. (2004) Brief but Efficient: Acute HIV Infection and the Sexual Transmission of HIV. The Journal of Infectious Diseases 189: 1785-1792.

46. Zaric GS, Barnett PG, Brandeau ML (2000) HIV transmission and the cost-effectiveness of methadone maintenance. American Journal of Public Health 90: 1100-1111.

47. Battegay M, Nüesch R, Hirschel B, Kaufmann GR (2006) Immunological recovery and antiretroviral therapy in HIV-1 infection. Lancet Infect Dis 6: 280-287.

48. Gazzola L, Tincati C, Bellistrı GM, d’Arminio Monforte A, Marchetti G (2009) The Absence of CD4+ T Cell Count Recovery Despite Receipt of Virologically Suppressive Highly Active Antiretroviral Therapy: Clinical Risk, Immunological Gaps,and Therapeutic Options. Clinical Infectious Diseases 48.
